# Stress, Life History, and Linear Enamel Hypoplasia: Insights From the Indigenous Populations of the Canary Islands

**DOI:** 10.1002/ajpa.70116

**Published:** 2025-08-29

**Authors:** Aarón Morquecho Izquier, Rebeca García‐González, Jonathan Santana

**Affiliations:** ^1^ G. I. Tarha. Departmento de Ciencias Históricas Universidad de Las Palmas de Gran Canaria Las Palmas de Gran Canaria Spain; ^2^ Laboratorio de Evolución Humana, Departamento de Historia, Geografía y Comunicación Universidad de Burgos Burgos Spain

**Keywords:** enamel hypoplasia, genetic diversity, island archeology, plasticity/constraint hypothesis, predictive adaptive response hypothesis

## Abstract

**Objectives:**

This study evaluated the influence of genetic diversity, subsistence strategies, age at death, and their interplay on the prevalence of linear enamel hypoplasias (LEHs) in the indigenous populations of the Canary Islands. Additionally, we test the predictive adaptive hypothesis and the plasticity/constraint hypothesis within this unique archeological context.

**Methods:**

LEH incidence, age of occurrence, and the number of stress episodes were assessed macroscopically in a sample of 409 individuals from six of the seven islands comprising the Canarian archipelago during the pre‐contact or Indigenous period (2nd–15th century cal CE). Statistical comparisons were made using chi‐square and Fisher's exact tests to evaluate LEH prevalence across populations and age groups within each island. To control for potential demographic confounding, hierarchical log‐linear (HLL) analysis was applied to explore the combined influence of age, sex, and island of origin on LEH prevalence. Model fit was assessed using likelihood‐ratio chi‐square tests.

**Results:**

Statistically significant differences were found between Gran Canaria and Tenerife, and between them and La Palma. Sexual differences in LEH prevalence were observed among individuals from Gran Canaria and within specific age groups in the other populations. In all indigenous populations, the number of individuals with LEH decreased in the oldest age groups.

**Conclusions:**

Our findings suggest that subsistence strategies explain the differences observed among the islands in terms of the various analyzed variables. All the data suggest that the plasticity/constraint hypothesis best fits the Indigenous populations of the Canary Islands, with males being more affected by environmental conditions than females.

## 1 Introduction

Enamel hypoplasia is a dental abnormality characterized by incomplete or defective mineralization of tooth enamel caused by a reduction in or interruption of ameloblast activity during the secretory stage of enamel formation (Guatelli‐Steinberg 2015; Towle and Irish 2020). This condition results in abnormal grooves and furrows on tooth crowns (Hillson 2014), which are typically classified into three categories: pits, planes, and linear forms (Guatelli‐Steinberg 2015). These different types of enamel hypoplasia arise from various causes, including malnutrition, disease, and trauma during tooth development (Guatelli‐Steinberg et al. 2007; Wilson 2014; Lawrence et al. 2021; Towle and Irish 2020).

Specifically, linear enamel hypoplasia (LEH) is often used as a permanent indicator of a disruption to the physiological homeostasis of the body that cannot be mitigated by cultural or biological mechanisms during a specific development period (Goodman et al. 1988). For this reason, the prevalence, number, and duration of LEH are extensively used in bioarcheological research to explore their associations with various factors, including subsistence strategies (Cohen 1989; Cohen and Armelagos 1984; Cucina 2002; Larsen 2006; Morquecho Izquier et al. 2023; Starling and Stock 2007; Steckel et al. 2002; Temple 2007, 2010, 2016), age at death (Cucina 2002; Duray 1996; Goodman et al. 1980; Palubeckaite et al. 2002; Saunders and Keenleyside 1999; Stodder 1997; Wyatt et al. 2023), sex‐based differences (Duray 1996; Lanphear 1990; Lovell and Whyte 1999; van Gerven et al. 1990) and interpopulation variability (Hutchinson and Larsen 1988; King et al. 2005; Wood 1996). Another important aspect of LEH research is that kinship and shared environmental conditions can also shape LEH prevalence and frequency. This consideration is particularly critical in archeological assemblages, where genetic relatives and household groups are often overrepresented (Lawrence et al. 2021). Unfortunately, the influence of genetic factors on LEH prevalence and their interaction with environmental factors cannot typically be assessed in archeological samples because of the lack of information about genetic background and similarity among individuals.

In addition to its utility as a physiological stress marker, LEH research provides a valuable framework for examining two key hypotheses regarding biological responses to early‐life stressors: the predictive adaptive response and the plasticity/constraint models within human life history theory. The predictive adaptive response, also known as the thrifty phenotype model, posits that mismatches between environmental conditions during fetal development and the postnatal environment trigger physiological adaptations that increase survival during early development but may have negative effects later in life if environmental conditions change (Gluckman, Cutfield, et al. 2005; Gluckman, Hanson, et al. 2005; Gluckman et al. 2007). In contrast, the plasticity/constraint hypothesis suggests that energetic investments in maintenance during early life may delay or inhibit future allocations to growth and reproduction, reflecting life history trade‐offs (Charnov 1991, 1993).

Studies have shown that individuals with LEH tend to have lower average ages at death than those without LEH (Cook and Buikstra 1979; Goodman and Armelagos 1988). These findings support the plasticity/constraint model, which links early‐life stress to reduced physiological resilience later in life. However, most of these studies focused solely on LEH incidence, overlooking the age of first occurrence and its relationship with age at death and the number of LEH events. These elements are essential for distinguishing between the predictive adaptive hypothesis and the plasticity/constraint hypothesis. For example, if no relationship is observed between the age of first occurrence, age at death, and number of LEH events, the plasticity/constraint model would be rejected in favor of the predictive adaptive hypothesis (Temple 2014). To address these questions, research must determine the age of LEH formation via macroscopic or microscopic methods (Amoroso et al. 2014; Wilson 2014). Additionally, interpretations should consider the influence of cultural, ecological, and biological factors, including sex differences.

**FIGURE 1:**
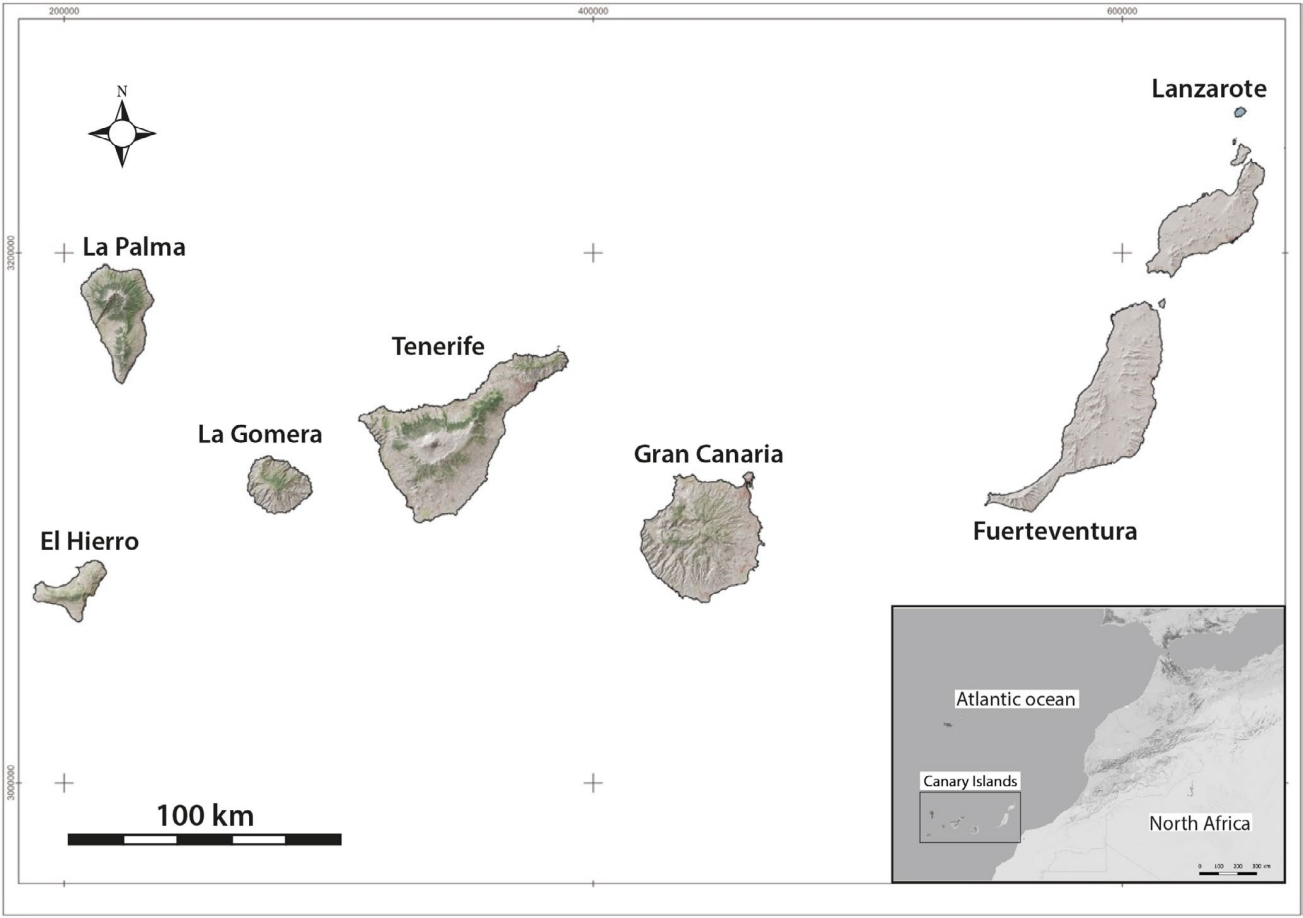
Map of the Canary Islands. The islands are located off the coast of western North Africa.

The indigenous period of the Canary Islands provides an ideal context for investigating these dynamics. This archipelago is composed of seven islands off the southern coast of northwestern Africa (Figure 1). Canary Island was first settled by Berber (Amazigh) populations between the first and third centuries CE (Santana et al. 2024) and developed distinct subsistence strategies and genetic profiles due to minimal interisland contact after initial settlement (Hagenblad et al. 2024; Morales et al. 2023). Differences in agriculture, livestock farming, and marine exploitation varied significantly across the islands, reflecting ecological and cultural adaptations.

For example, agriculture was dominant on Gran Canaria and important on Tenerife, but declined on La Gomera and El Hierro and was abandoned on La Palma and Fuerteventura (Arnay‐de‐la‐Rosa et al. 2010; Delgado‐Darias et al. 2005; Morales et al. 2017, 2023; Santana et al. 2015). Livestock farming, particularly goat milk production, is important across islands but has been less studied on Gran Canaria Island (Arnay‐de‐la‐Rosa et al. 2009, 2011, 2010; Brito‐Mayor et al. 2023; Sánchez‐Cañadillas et al. 2023, 2021). The exploitation of wild animals, especially in Tenerife and Fuerteventura, has caused species extinction (de Nascimento et al. 2020; Morales et al. 2009; Rando et al. 2012). Marine resources are vital for Gran Canaria, La Palma, Tenerife, and El Hierro, with El Hierro showing greater reliance on marine products than Tenerife does (Mesa Hernández 2016, 2018; Parker et al. 2020; Rodríguez Santana 1994; Rodríguez‐Rodríguez et al. 2021; Sánchez‐Cañadillas et al. 2023).

Additionally, archeological and genetic evidence indicate differences in genetic diversity and demographic growth over time between islands (Serrano et al. 2023; Velasco‐Vázquez et al. 2021). Gran Canaria, Tenerife, and La Palma have experienced demographic expansion over time, which has helped maintain genetic diversity since the early stages of island settlement. In contrast, El Hierro, La Gomera, Lanzarote, and Fuerteventura presented clear signs of strong genetic drift, leading to a gradual reduction in their effective population sizes. By the 10th–12th centuries CE, evidence suggests that some individuals from these islands resulted from close‐kin matings, which is consistent with the reduced population sizes during this period (Serrano et al. 2023).

These factors make the Canary Islands a valuable case for exploring how genetic diversity, subsistence strategies, and their interplay influence LEH prevalence, timing, and implications for demographic dynamics and mortality. Thus, the primary objective of this study was to explore the influence of genetic diversity, subsistence strategies, age at death, and their interplay on the prevalence, age at first occurrence, and frequency of LEHs in indigenous populations of the Canary Islands. Specifically, the research aims to test the applicability of the predictive adaptive hypothesis and the plasticity/constraint hypothesis within this unique archeological context. To achieve this goal, we analyze LEH data in relation to age at death, sex, and subsistence practices across different islands, integrating archeological, ecological, and genetic evidence. By examining these factors, this study seeks to provide a comprehensive understanding of the long‐term impacts of early‐life stress on mortality, contributing to broader discussions on human adaptability and life history strategies in bioarcheology.

## 2 Materials and Methods

### 2.1 Materials

This study examines a large assemblage of skeletonized individuals from indigenous populations of the Canary Islands. A total of 409 adult individuals from six of the seven islands were evaluated (Table 1). These human remains are stored in island archeological museums (Canary Islands) and the Musée de l'Homme in Paris (France). Most remains were collected between the late 19th century and the early 20th century by Canarian and European anthropologists. This study also incorporates human remains from more recent archeological excavations of the 20th and 21st centuries.

**TABLE 1:** Demographic profile of each of the Canarian indigenous populations.

Population	Female					Male					Unknown^a^	Total
Age^b^	17–25	26–35	36–45	> 45	Total	17–25	26–35	36–45	> 45	Total		
El Hierro	5	2	3	3	28	9	6	11	5	40	30	98
La Palma	1	2	4	0	7	4	4	2	3	15	14	36
La Gomera	9	3	0	0	16	13	5	2	0	28	21	65
Tenerife	3	2	0	0	9	4	5	2	0	15	6	30
Gran Canaria	13	16	5	1	52	8	17	6	0	58	48	158
Fuerteventura	5	0	1	0	6	9	2	1	0	13	3	22
Total	36	25	13	4	118	47	39	24	8	169	122	409

^a^ Unknown individuals are those for whom a sex and age estimation could not be performed.

^b^ Age in years.

The samples included in this study span a broad temporal range, covering the period between the initial Amazigh colonization of the Canary Islands and their European conquest (Table 2). While this timeframe is indeed extensive (ca. 2nd–15th centuries CE), multiple lines of archeological, genetic, and linguistic evidence suggest a relatively rapid and synchronous colonization of the archipelago, likely completed within approximately 200 years during the early centuries CE (Santana et al. 2024). We acknowledge that this long timeframe introduces potential for temporal bias. However, direct chronological assessment of individual remains is limited by funding constraints and museum policies that restrict destructive sampling, precluding the systematic use of radiocarbon dating for all individuals. As a result, we were unable to construct precise chronological groups for analysis.

**TABLE 2:** Estimates of initial colonization and dates of European conquest for each island derived from Santana et al. (2024).

Island	Chronology	
	Amazigh colonization (CE)	European conquest (CE)
El Hierro	170–330	1405
La Palma	245–430	1494
La Gomera	275–404	1488
Tenerife	155–485	1496
Gran Canaria	490–530	1486
Fuerteventura	270–525	1402

Despite this limitation, the overall chronological span of the samples from each island is broadly similar (see Table 2), with all islands represented primarily by remains dating from the early first millennium CE to the period immediately before European contact. This reduces the likelihood that the observed inter‐island differences in LEH prevalence or other covariates are driven solely by chronological variation.

### 2.2 Sex and Age Estimations

The sex estimation was primarily based on cranial morphology (Buikstra and Ubelaker 1994), as most individuals lacked postcranial elements. However, when the pelvic remains were present, they were used for sex determination. For age estimation, we applied Brothwell's (1981) dental wear method, acknowledging that this method can be influenced by factors other than age. In the case of the Gran Canaria population, the age ranges estimated with this method align with those obtained from pubic symphysis and auricular surface changes (Delgado‐Darias et al. 2005, 2006). However, we lack evidence of such consistency for other populations. There are differences in dental macrowear across populations, largely attributed to variations in subsistence strategies (Morquecho Izquier et al. 2025), which may affect the comparability of age ranges between populations. For example, El Hierro and La Palma presented relatively high levels of heavy dental macrowear (Tables S1–S4), which might explain why individuals over 45 years of age were more commonly identified in these populations (Table 1). We took these aspects into account when processing the data and interpreting our results.

### 2.3 LEH Prevalence

LEH is usually assessed on incisors and canines, as these are the teeth with the highest likelihood of exhibiting enamel hypoplasia (Goodman and Armelagos 1985; Hillson 1996). However, those teeth are the least represented in the sample (Table 3).

**TABLE 3:** Tooth class distribution in the Canarian Indigenous population.

Tooth class	N	%
Incisors	359	11.9
Canines	245	8.1
Premolars	724	24.0
Molars	1692	56.0
Anterior	604	20.0
Posterior	2416	80.0
Total	3020	100

This sample bias stems from the characteristics of the osteological collection, which originates from explorations conducted in the 19th to 20th centuries by Canarian and European anthropologists. These explorations lacked archeological methodology and focused primarily on the skull and long bone remains for osteometric analyses. Consequently, the retrieval methods were not designed to collect all the osteological evidence, resulting in a bias in the dental representation. When the periodontal ligament decays, single‐root teeth are prone to fall out when the cranium and mandible are displaced. In contrast, teeth with two or three roots tend to still be within the alveoli (Duday 2009). Consequently, if we focused only on the anterior teeth, we would not only significantly reduce the sample size but also have very few cases where we could confidently determine that they belong to the same individual. As we explain below, this last point is crucial when interpreting the results of LEH prevalence.

Thus, we only scored LEH on the first and second molars, both maxillary and mandibular. To do that, dental remains were examined via a macroscopic visual inspection method aided by a 10× magnifying lens under both natural and artificial lighting conditions. The identification of enamel defects followed the established method described by Goodman and Rose (1990), who classified them as pits and lines. However, for the calculation of LEH incidence, only linear defects were considered for this study. The evaluation of enamel defects revealed three outcomes: absent, present, or not observable.

An individual was considered to have LEH when defects were identified in at least two isomeres or antimeres following the criteria set by Ham et al. (2021) and Klaus and Tam (2009). This more stringent criterion for categorizing an individual in the collection as affected by LEH aims to ensure that these defects are attributed to physiological stress episodes rather than traumatic injuries or congenital defects (Goodman and Rose 1990; Hillson and Bond 1997; Nikiforuk and Fraser 1981). LEH is determined by the ratio of affected individuals to those for whom LEH could be confidently assessed. We acknowledge that in bioarcheology, this does not reflect true prevalence in the epidemiological sense, as we cannot know the total number of individuals at risk in the original population. However, in keeping with standard practice in the field, we use the term *prevalence* to refer to this ratio, while recognizing its limitations.

We acknowledge that using a macroscopic approach based on molars, especially in populations with significant differences in dental macrowear, could impact our results. Given the low resolution of macroscopic methods, LEHs are more easily detected where perikymata are more widely spaced or where longer periods of stress affect multiple perikymata (Cares Henriquez and Oxenham 2017). The spacing of perikymata depends on the way in which dental tissues are deposited (Smith 2008). Within the same tooth, this spacing depends on the number of perikymata and on the extension rate. The density of perikymata increases progressively toward the cervix of the crown, with the highest number of perikymata per decile observed in the final 20% of crown height across all teeth (Dean and Reid 2001; Reid and Dean 2006).

The extension rate can vary along the entire height of the crown, leading to both differences in the spacing of perikymata and the angle of striae emergence (Guatelli‐Steinberg et al. 2012; Hillson and Bond 1997). In molars, perikymata are more widely spaced in the more occlusal areas, and in the intermediate regions, they are shallower due to the striae's acute angle of emergence of perikymata (Guatelli‐Steinberg et al. 2012; Guatelli‐Steinberg and Reid 2008; Hillson and Bond 1997). Moreover, molars hold a larger proportion of cuspal enamel (where perikymata are not visible), which, alongside the effect of macrowear, can make it challenging to detect LEH in the occlusal areas. To account for these factors in our results, we divided the crown into three different developmental parts: occlusal, intermediate, and cervical. The occlusal region includes both the cusp tip and the adjacent lateral enamel surface. Although this region corresponds to the earliest stages of crown formation, LEH defects can still be observed here when formed early in development. In the occlusal part, we can evaluate the impact of the large proportion of cuspal enamel of molars and varying degrees of macrowear light, medium, and heavy on LEH detection. In the intermediate portion, we can evaluate the effects of the angle of emergence of perikymata alongside the medium and heavy degrees of macrowear. Finally, in the cervical region, we can assess LEH detection where perikymata are tightly packed.

To accomplish this, we first determined the position of the LEH on the tooth crown as the distance from the cementoenamel junction (CEJ). Second, to establish in which developmental part each LEH was situated, we divided the crown height into three parts and compared the distance from the CEJ to the boundaries of these three crown regions. To establish these boundaries, we used the population‐specific average crown heights. A pooled sex mean was used, as sex could not be determined for many individuals (Tables 1, S5, and S6). For Tenerife, where lower molar data were limited, the average across all indigenous populations was used. This assumption is supported by earlier findings that demonstrated a consistent dental size pattern among the indigenous population, characterized by inferior and superior molars with proportionate dimensions (Irish and Hemphill 2004). Since no statistically significant differences were found in the size of the upper molars in Tenerife compared with other populations (García‐González et al. 2025), we can expect that there are also no statistically significant differences in the lower molars.

### 2.4 Age at Occurrence of LEH and Number of Stress Episodes

There are two main methods used to estimate the age of LEH formation: counting perikymata and measuring distances from LEH to the incisal/occlusal edge (Smith 2008). The first method was not feasible because of the visibility of perikymata. The second method involves measuring distances and comparing them with dental development charts, but it is challenging with worn teeth, as it requires crown reconstruction. Various methods for reconstructing worn teeth have yielded accurate results (Guatelli‐Steinberg and Reid 2008; Modesto‐Mata et al. 2017; O'Hara and Guatelli‐Steinberg 2022; Saunders et al. 2007; Smith et al. 2012). However, the accuracy of these methods depends on their application to slightly worn teeth and/or the use of micro‐CT slices. Owing to constraints in applying reconstruction methods, we chose not to reconstruct crown heights. Instead, we measured the position of the top of each LEH from the CEJ with a sliding caliper (Cucina 2002). Since this measurement does not follow the growth direction of enamel, an additional step is needed before transformation into chronological age, which considers the total crown height. As in the case of the developmental parts explained above, we used population‐specific average crown heights. In this case, instead of dividing the crown height into three parts, we divided it into 10 parts (deciles). Reid and Dean (2006) proposed a standard for the age of attainment of each of these deciles for North European and South African populations.

To estimate the age interval during which each LEH episode likely occurred, we compared their position to the boundaries of these deciles provided by Reid and Dean (2006). These authors proposed two different standards for the age of attainment of each of these deciles, one for North Europeans and the other for South Africans. The main difference between these two populations is in the formation times of the first molars. Paleogenomic studies indicate that the Canary Islands were settled by autochthonous northwestern African groups (Fregel et al. 2019; Serrano et al. 2023). Dhamo et al. (2018) demonstrated that southern Africans experience faster dental development than northern Europeans do. Consequently, for Canarian indigenous individuals, the Southern African standards of Reid and Dean (2006) were deemed more appropriate. On the basis of this approach, the ages of occurrence of LEH were grouped into four age groups. These age groups were established considering two aspects. The first factor is the age of cusp completion and crown completion of the first and second molars for South Africans. Cusp completion occurs over 1 year for the first molar and approximately 4 years for the second molar (Reid and Dean 2006). Crown completion takes place approximately 3.5 years for the first molar and approximately 6 years for the second molar (Reid and Dean 2006).

LEH can be detected only when it occurs between 1 and 3.5 years in the first molar and between 4 and 6 years in the second molar. The second factor considered in the establishment of the groups was the metabolic demands of growing children and the maturation of the immune system. The greatest metabolic demand during early childhood occurs within the first 2 years of life and is driven primarily by the brain's metabolic requirements (Leonard et al. 2007). During this period, a significant portion of these metabolic needs is met by the infant's fat stores, most of which accumulate during gestation (Kuzawa 1998; Leonard et al. 2007). Furthermore, the immune system remains immature during the first 2 years of life (Bogin 1999). Therefore, for up to 2 years, the presence of LEH may be related to this immature system of children and limited fat stores. From ages 2 to 5, individuals still have a relatively large, metabolically expensive brain, but their fat stores rapidly decrease. As a result, children require a nutrient‐dense diet, although they have not yet developed the skills to acquire a diet on their own (Bogin 1999). Therefore, disparities in LEH prevalence from 2 to 55 years of age may be interpreted as reflections of variation among populations in terms of access to vital resources, encompassing differential levels of parental care, nutrition, and healthcare.

In summary, we established the following age groups for the occurrence of LEH: (1) 1–2, (2) 2–3.5, (3) 4–5, and (4) 5–6 years. In the first two groups, LEH was detected in the first molar, and in the second two groups, it was detected in the second molars. Specifically, for the first molar, LEH defects located up to decile 4 were assigned to the 1‐ to 22‐year group, while those from decile 5 onward were assigned to the 2‐ to 3.55‐year group. For the second molar, defects up to decile 5 were classified as occurring between 4 and 55 years, and those from decile 6 were assigned to the 5‐ to 66‐year group. This approach allowed us to group LEH occurrences according to meaningful stages of dental development while minimizing overlap between age categories.

The assessment of multiple LEH episodes was conducted by ensuring that there was no overlap in the age intervals for each LEH detected, thereby increasing the accuracy of the timing of stress episodes.

### 2.5 Sources of Variation in LEH Prevalence

We performed the following analyses: (1) comparisons of LEH prevalence across the comprehensive sample from each island population and (2) analysis of LEH prevalence segmented by sex, interval of age of occurrence of LEH, and age‐at‐death groups in each population. In both cases, chi‐square tests and Fisher's exact tests were employed for these comparisons, with a predefined confidence threshold of 95% (*p* < 0.05). We refrained from comparing age groups between populations and instead focused on comparing LEH prevalence among age groups within the same island. This approach allows us to detect intrapopulation differences among age groups, even though we cannot be certain whether these age estimates are consistent with other skeletal indicators.

To evaluate whether inter‐island differences in LEH prevalence could be attributed to variation in the demographic composition of the samples—specifically, sex or age‐at‐death distributions—we first conducted chi‐square tests comparing the frequency distributions of these variables across islands. These comparisons were performed both for the full six‐island data set and for the subset of islands in which statistically significant differences in LEH prevalence were identified.

In cases where chi‐square tests indicated significant variation in sex or age distributions, we applied hierarchical log‐linear (HLL) analysis to assess the joint association between LEH prevalence, demographic variables (age group or sex), and island of origin (Yaussy and DeWitte 2018). HLL is a multivariate statistical technique that allows the simultaneous evaluation of relationships among three or more categorical variables, making it particularly useful when potential confounding is present. Unlike standard chi‐square tests, which assess associations between two variables at a time, HLL can detect both independent effects and interaction effects.

The analysis proceeds by fitting a sequence of nested models, beginning with one that assumes complete independence among variables, and gradually introducing interaction terms. Model fit is assessed using likelihood‐ratio chi‐square tests, which indicate whether the addition of interaction terms significantly improves the model. A model that includes significant interaction terms suggests that the association between LEH and island cannot be fully explained by age or sex independently, but instead reflects a combined effect. Model fit was evaluated using likelihood‐ratio chi‐square tests, and ANOVA comparisons between models were used to assess whether the inclusion of interaction terms significantly improved the fit. A significant reduction in deviance after adding interaction terms suggests that the distribution of LEH cannot be explained by the independent effects of age (or sex) and island alone, but rather by their combined influence. This approach allows us to account for potential confounding when evaluating inter‐island differences in LEH prevalence.

## 3 Results

### 3.1 Evaluating the Effect of Dental Wear in the Detection of LEH

**FIGURE 2:**
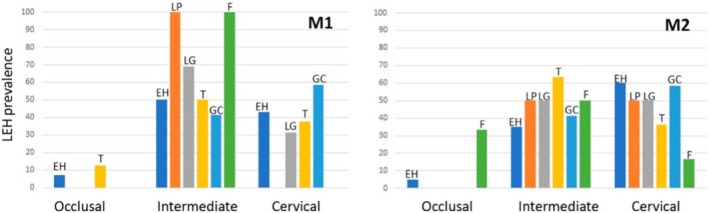
Distribution of LEH in the occlusal, intermediate, and cervical regions of the first and second molars by each island. EH, El Hierro; F, Fuerteventura; GC, Gran Canaria; LG, La Gomera; LP, La Palma; T, Tenerife.

Given that our results may be influenced by differences in attrition between populations, we considered plotting the distribution of LEH in the occlusal, intermediate, and cervical regions of the first and second molars (Figure 2). In both teeth, the number of LEHs detected was minimal in the cuspal third, with most being detected in the intermediate and cervical thirds. Despite variations in macrowear among populations, LEH has rarely been detected in the occlusal portion of the crown. In the occlusal third, LEH appeared only in El Hierro (M1, M2), Tenerife (M1), and Fuerteventura (M2). Interestingly, El Hierro and Tenerife, despite high levels of macrowear, presented LEH in the occlusal third, whereas Fuerteventura's lower macrowear may explain its detection there. Overall, interpopulation differences had little impact on LEH prevalence. Instead, the low resolution of the macroscopic method likely hindered defect detection in the occlusal third because of shallow, poorly defined perikymata. However, this limitation affected all populations similarly, resulting in comparable LEH prevalence rates.

### 3.2 LEH Prevalence by Population, Sex, and Age at Death Group

Table 4 displays the LEH prevalence at the individual level for each population and sex. Significant differences in LEH prevalence were observed between the populations of La Palma and Tenerife (Table S7, odds ratio = 0.19, *p* = 0.01) and between those of La Palma and Gran Canaria (Table S7, odds ratio = 0.30, *p* = 0.03). In both cases, La Palma presented a lower LEH prevalence.

**TABLE 4:** Presence of LEH and prevalence (%) in the Canarian indigenous population at the individual level by island and sex.

Population	Female	Male	Total
El Hierro	9/28 (32.14%)	9/40 (22.50%)	24/98 (24.49%)
La Palma	1/7 (14.28%)	2/15 (13.33%)	4/36 (11.11%)
La Gomera	4/16 (25.0%)	7/28 (25.0%)	18/65 (27.6%)
Tenerife	4/9 (44.44%)	6/15 (40.0%)	12/30 (40.0%)
Gran Canaria	**10/52 (19.23%)**	**22/58 (37.93%)**	46/158 (29.11%)
Fuerteventura	3/6 (50.0%)	4/13 (30.77%)	7/22 (31.82%)
Total	31/118 (26.27%)	50/169 (29.58%)	111/409 (27.13%)

*Note:* The bold font indicates statistically significant differences.

Sexual differences in each of the populations were found only in the Gran Canaria population (Table S8, chi‐square = 4.64, *p* = 0.04), where females had a significantly lower LEH incidence than males.

**FIGURE 3:**
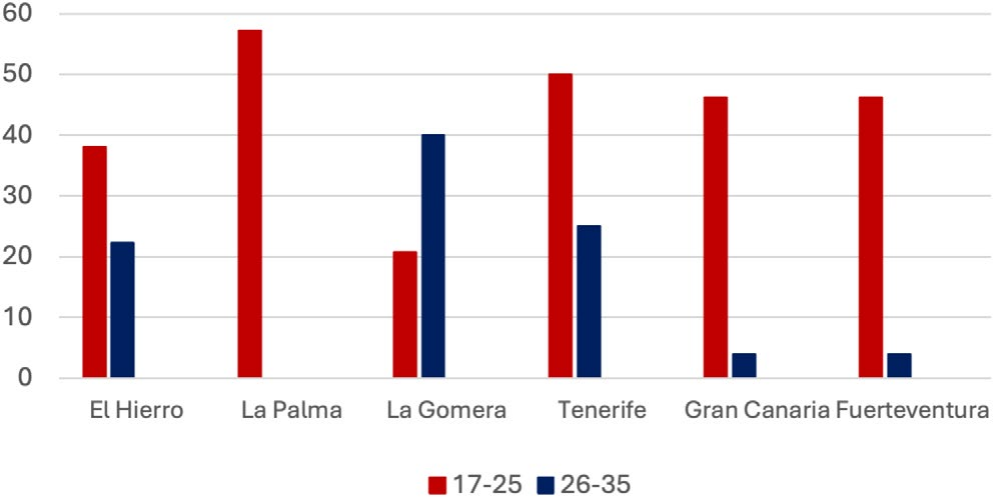
LEH prevalence by age group and island population. The *Y*‐axis shows the LEH prevalence within the age group.

Figure 3 depicts the frequency of LEH for each age group and population. None of the older age groups showed evidence of LEH, and with the exception of La Gomera, the LEH prevalence was higher in the 17‐ to 255‐year age group than in the 26‐ to 355‐year age group. The change in LEH frequency between these two age groups was statistically significant only in La Palma and Gran Canaria (Table S9).

To rule out confounding by unequal sex ratios, we first compared male/female counts across all six islands. A chi‐square test showed no significant differences (*χ* ^2^ = 3.76, df = 5, *p* = 0.584), and the same held when restricting to La Palma, Tenerife, and Gran Canaria (*χ* ^2^ = 2.99, df = 2, *p* = 0.224). Thus, sex‐ratio variation is unlikely to account for the observed LEH differences.

By contrast, age‐group distributions did differ significantly among islands (*χ* ^2^ = 58.32, df = 15, *p* < 0.001) and remained so within the three‐island subset (*χ* ^2^ = 13.97, df = 6, *p* = 0.030; Fisher's exact *p* = 0.055). These results suggest that age structure varies across islands and may influence LEH frequencies. Indeed, the HLL analysis revealed a statistically significant three‐way interaction between LEH, age group, and island (Table 5). This result indicates that the association between LEH prevalence and island varies across age groups and cannot be fully explained by the independent or pairwise effects of these variables alone.

**TABLE 5:** Likelihood‐ratio tests comparing hierarchical log‐linear models of LEH prevalence, age group, and island or origin.

	Model 1	Model 2
	Two‐way interaction	Three‐way interaction
Deviance	34.76	0.00
df	15	0
ΛDeviance	…	34.76
Λdf	…	15
*p*‐value	…	0.03

*Note:* Model 1 includes only two‐way interactions among LEH status (present or absent), age‐at‐death group, and island. Model 2 includes the full three‐way interaction among these variables. Deviance represents the difference between observed and expected frequencies under the model. Degrees of freedom (df), change in deviance (ΔDeviance), and change in degrees of freedom (Δdf) are reported to compare model fit. The *p* value derives from a likelihood ratio test evaluating whether the three‐way interaction in Model 2 significantly improves model fit. A significant *p* value indicates that the association between LEH status and island differs by age‐at‐death group.

### 3.3 Age at Occurrence of LEH and Number of Episodes of Stress

The number of LEHs formed at each age of occurrence group was consistent among the different indigenous populations (Table 6). When comparing populations within these age groups, statistical analyses revealed no significant differences (Tables S10 and S11).

**TABLE 6:** Percentages of LEH by age of formation, sex, and population.

Population	Sex	1–2 years old	2–3.5 years old	4–5 years old	5–6 years old
El Hierro	Female	33.33% (3/9)	22.22% (2/9)	11.11% (1/9)	77.78% (7/9)
	Male	33.33% (3/9)	22.22% (2/9)	11.11% (1/9)	66.67% (6/9)
	Total	29.17% (7/24)	25.0% (6/24)	12.50% (3/24)	66.67% (16/24)
La Palma	Female	100% (1/1)	…	100% (1/1)	…
	Male	100% (2/2)	…	100% (2/2)	…
	Total	75% (3/4)	…	100% (4/4)	…
La Gomera	Female	25.0% (1/4)	25.0% (1/4)	…	100% (4/4)
	Male	42.86% (3/7)	28.57% (2/7)	14.29% (1/7)	85.71% (6/7)
	Total	50.0% (9/18)	16.67% (3/18)	11.11% (2/18)	83.33% (15/18)
Tenerife	Female	25.0% (1/4)	50.0% (2/4)	…	75.0% (3/4)
	Male	33.33% (2/6)	16.67% (1/6)	…	83.33% (5/6)
	Total	33.33% (4/12)	25.0% (3/12)	…	91.67% (11/12)
Gran Canaria	Female	50.0% (5/10)	80.0% (5/10)	10.0% (1/10)	80.0% (8/10)
	Male	27.27% (6/22)	45.45% (10/22)	31.82% (7/22)	77.27% (17/22)
	Total	34.8% (16/46)	56.5% (26/46)	21.7% (10/46)	73.9% (34/46)
Fuerteventura	Female	33.33% (1/3)	66.67% (2/3)	…	33.33% (1/3)
	Male	25.0% (1/4)	…	75.0% (3/4)	50.0% (2/4)
	Total	28.57% (2/7)	28.57% (2/7)	42.86% (3/7)	42.86% (3/7)

*Note:* The sum of individuals in each age group is greater than the total number of individuals, as there are individuals who experience more than one event of stress.

Sexual differences were found in the 5‐ to 6‐year‐old age range in La Gomera and Tenerife (*p* = 0.01 in both cases) and in the 4‐ to 5‐year‐old age range in Gran Canaria (*p* = 0.05). In Tenerife and Gran Canaria, the number of LEHs formed in each of these age ranges was significantly lower in females than in males, whereas the opposite was true in La Gomera (Tables 6 and S12).

Table 7 presents the number of individuals with one, two, or three stress episodes in each indigenous population.

**TABLE 7:** Number of stress episodes by population and sex.

Population	Sex	One episode	Two episodes	Three episodes
El Hierro	Female	55.56% (5/9)	33.33% (3/9)	11.11% (1/9)
	Male	77.78% (7/9)	11.11% (1/9)	…
	Total	70.83% (17/24)	25.0% (6/24)	4.17% (1/24)
La Palma	Female	…	…	100% (1/1)
	Male	…	100% (2/2)	…
	Total	25.0% (1/4)	50.0% (2/4)	25.0% (1/4)
La Gomera	Female	50.0% (2/4)	50.0% (2/4)	…
	Male	42.86% (3/7)	57.14% (4/7)	…
	Total	38.89% (7/18)	44.44% (8/18)	11.11% (2/18)
Tenerife	Female	50.0% (2/4)	50.0% (2/4)	…
	Male	50.0% (3/6)	33.33% (2/6)	16.67% (1/6)
	Total	53.85% (7/13)	30.77% (4/13)	7.69% (1/13)
Gran Canaria	Female	20.0% (2/10)	40.0% (4/10)	40.0% (4/10)
	Male	31.82% (7/22)	45.45% (10/22)	13.64% (3/22)
	Total	30.43% (14/46)	47.83% (22/46)	19.57% (9/46)
Fuerteventura	Female	66.67% (2/3)	33.33% (1/3)	…
	Male	50.0% (2/4)	50.0% (2/4)	…
	Total	57.14% (4/7)	42.86% (3/7)	…

El Hierro, Tenerife, and Fuerteventura are notable for having a greater number of individuals showing only a single stress event. However, significant differences were observed only when El Hierro was compared with Gran Canaria (Table S14). With respect to individuals with two stress episodes, there was notable consistency across populations, with no statistically significant differences between them (Tables 7 and S15).

There are generally fewer individuals who experienced three stress episodes across all populations. An exception to this trend is observed in La Palma, where the number of individuals with one stress episode is equal to those experiencing three stress episodes. Despite Gran Canaria and La Palma having the highest counts of individuals with three stress episodes, these numbers do not differ significantly from those of other island populations (Tables 7 and S16). This suggests that while there are variations in the number of stress episodes experienced by individuals within populations, these differences are not always statistically significant when compared across different island populations.

## 4 Discussion

This study revealed that the LEH prevalence in the indigenous population from the Canary Islands ranges from 11.11% to 40% (Table 4), which is lower than that reported in populations with poor quality of life indices (Ham et al. 2021; Yaussy et al. 2016). However, when comparing LEH frequencies across studies, it is important to consider the type of teeth analyzed. Most research reporting higher prevalence rates focuses on anterior teeth, particularly permanent incisors and canines, which are more susceptible to hypoplastic defects, have thinner enamel, and exhibit more distinct perikymata, making LEH easier to identify (Goodman and Rose 1990; Hillson 1996; Lukacs 1989). In contrast, the present study is based on first and second molars, which generally show lower frequencies of LEH primarily due to the difficulty of detecting subtle defects on their thicker enamel surfaces and the higher proportion of cuspal enamel (Guatelli‐Steinberg 2001).

The higher proportion of cuspal enamel may help explain why most LEH defects were detected in the intermediate and cervical thirds, with only minimal occurrences in the occlusal third. While LEH in the occlusal third was observed in El Hierro (M1, M2), Tenerife (M1), and Fuerteventura (M2), the overall rarity of defects in this area, despite differing levels of macrowear, suggests a methodological limitation rather than a biological pattern. Notably, El Hierro and Tenerife showed LEH in the occlusal third despite higher levels of wear, while its presence in Fuerteventura may be attributed to lower occlusal wear. These findings suggest that interpopulation differences in wear had a limited influence on LEH detection. Instead, the low resolution of macroscopic inspection methods—particularly in the occlusal third, where perikymata are shallow and less defined—likely hindered defect identification uniformly across populations. Consequently, the relatively low and comparable LEH prevalence observed here likely reflects both tooth‐type selection and methodological constraints, rather than indicating reduced physiological stress or improved health conditions in the studied populations.

Significant differences in LEH prevalence were identified between Tenerife and Gran Canaria, and between them and La Palma. These variations suggest that subsistence practices played a crucial role in the lower LEH prevalence in La Palma.

In Gran Canaria and Tenerife, agriculture was central to subsistence, whereas in La Palma, it became less significant around the 10th century CE during the Medieval Climate Oscillation (Morales et al. 2023). In response, La Palma intensified the use of livestock, wild plant resources, and marine foodstuffs (Arnay‐de‐la‐Rosa et al. 2010; Morales et al. 2017; Pais 1996; Pérez González et al. 2001; Rodríguez Santana 1994). Compared with Gran Canaria, this diversified subsistence strategy likely mitigated food shortages, reducing stress events during early development. In Gran Canaria, reliance on agriculture may have contributed to higher LEH prevalence, as cereal‐based systems are vulnerable to instability (Delgado‐Darias 2009). However, adaptations such as granaries and irrigation systems helped buffer these challenges, allowing demographic growth (Henríquez‐Valido et al. 2019; Morales and Gil 2014; Morales et al. 2023; Velasco‐Vázquez et al. 2021). Notably, Gran Canaria also presented the greatest number of individuals with two stress episodes, potentially reflecting repeated exposure to stress during early development.

Despite the shared demographic history of Gran Canaria and La Palma, which includes demographic expansion and the maintenance of genetic diversity from the early stages of settlement (Santana, del Pino Curbelo, et al. 2025; Serrano et al. 2023), these genetic similarities do not appear to significantly influence the observed differences in LEH prevalence between the two islands. While kinship and shared environmental conditions are recognized as factors that can shape LEH prevalence and frequency (Lawrence et al. 2021), the patterns identified in this study suggest that other variables, such as subsistence strategies and local environmental stressors, play a more prominent role. In the case of Gran Canaria and La Palma, the observed differences in LEH seem to reflect variations in adaptive responses to environmental stress rather than being directly attributable to genetic similarities. This highlights the importance of considering both biological and cultural factors when interpreting patterns of stress in past populations.

LEH was absent in individuals from the oldest age‐at‐death groups (> 36 years), suggesting that those who experienced early developmental disruptions had reduced chances of reaching older ages. Additionally, in La Palma and Gran Canaria, the LEH prevalence significantly decreased between the age‐at‐death groups of 17–255 years and the older groups. This further supports the idea that developmental disruptions negatively affect long‐term survival. These results align with many studies showing a direct relationship between LEH and reduced longevity (DeWitte and Wood 2008; Duray 1996; Goodman and Armelagos 1988; Hawks et al. 2022; King et al. 2005; Stodder 1997). However, variation in LEH prevalence between islands may also be partially influenced by differences in the age structure of the samples. Chi‐square tests revealed that age‐group distributions differ significantly among the six islands, and this difference persisted when restricting the analysis to the three islands among which we detected statistically significant differences (La Palma, Tenerife, and Gran Canaria). These results indicate that some of the observed inter‐island variation in LEH frequencies could reflect differing proportions of younger vs. older individuals in the samples, especially given that LEH tends to be more prevalent in individuals who died in early adulthood. Although we cannot rule out the possibility that the observed differences in age distributions are due to sampling biases, it is also important to consider that these variations may reflect actual demographic dynamics, such as differences in migration and fertility rates both within and between the populations studied (Sattenspiel and Harpending 1983; Buikstra et al. 1986; Wood et al. 1992).

Migration has been excluded as a significant factor in the population dynamics of the Canary Islands, as genetic studies have shown little exchange between islands after the initial colonization period (Fregel et al. 2019; Serrano et al. 2023). Instead, research reveals that climatic conditions played a more decisive role in shaping demographic trends (Santana, del Pino Curbelo, et al. 2025). Islands with more humid climates, such as Gran Canaria and Tenerife, experienced more favorable conditions for habitation, which facilitated sustained population growth. In contrast, the arid islands, particularly Lanzarote and Fuerteventura, faced agrarian challenges due to higher temperatures, erratic rainfall, and increased evaporation, all of which likely hampered crop production—a key factor in demographic stability (Santana, del Pino Curbelo, et al. 2025). These climatic differences, coupled with the biogeographical characteristics of each island, influenced the carrying capacities of the islands, with Gran Canaria and Tenerife exhibiting the most consistent population growth. Additionally, fertility patterns may have been affected by these environmental constraints, as more stable climates likely support higher fertility rates and greater population sustainability, while arid conditions could have imposed limitations on reproductive success and survival.

Bioarcheological analysis of post‐contact individuals from Early Modern Gran Canaria (16th–18th centuries) indicates a marked reduction in the prevalence of LEH compared to pre‐contact Indigenous populations (Morquecho Izquier et al. 2023). Stable isotope data and oral health suggest that this reduction is attributed to improved early‐life nutritional conditions. The gradual introduction of new crops, particularly maize, along with sugarcane and introduced animals such as cattle and chickens, resulted in more stable and calorically dense diets (Morquecho Izquier et al. 2023; Santana, Sánchez Cañadillas, et al. 2025). Advancements in agricultural technologies and integration into transatlantic trade networks also contributed to mitigating seasonal food scarcity. These transformations likely reduced the frequency and severity of physiological disruptions during enamel formation (Morquecho Izquier et al. 2023).

The results of the HLL further support an interpretation of the effect of the demographic dynamics in the differences in the LEH prevalence by age group, as age at death was a significant predictor of the likelihood of exhibiting LEH (Table 5). Specifically, individuals who died at younger ages were more likely to show signs of enamel hypoplasia, whereas older individuals had a lower probability of presenting visible defects. This finding is consistent with both biological expectations—since individuals exposed to stress early in life may have experienced reduced survivorship—and taphonomic or methodological biases, such as tooth wear and loss in older individuals. Therefore, the significant differences in age distributions between island samples could have influenced the observed variation in LEH prevalence, reinforcing the need to interpret inter‐island differences cautiously and in light of these demographic and preservation‐related factors.

Additionally, we must consider other alternative explanations reflecting taphonomic or methodological biases rather than actual differences in survival. LEH defects formed early in life can be gradually obliterated due to mastication, particularly in molars, where enamel loss is more pronounced over time (King et al. 2005). Additionally, there is a clear pattern of increasing prevalence of antemortem tooth loss across successive age groups on all islands (Morquecho Izquier et al. 2025). This result is expected in archeological populations, as the causes typically involved in tooth loss during life—caries, dental wear, or periodontal disease—are chronic in nature (Bonsall 2014). It may reduce the probability of observing LEH in the oldest age groups, which is especially relevant in this study because LEH prevalence was calculated as the proportion of affected individuals rather than being adjusted for the amount of observable enamel surface available. Consequently, individuals with LEH obliterated for wear may have been misclassified as unaffected. However, it is important to note that the occlusal third, where perikymata are more likely to be obliterated, was generally the least informative for LEH detection across all age groups and populations. As mentioned above, most defects were found in the middle and cervical thirds, which tend to be more resistant to wear. Therefore, while tooth wear and loss may partially explain the absence of LEH in older individuals, genuine differences in survivors seem to be the most plausible explanation.

Significant sex differences in LEH incidence were observed between individuals from Gran Canaria, with females showing a lower LEH incidence than males. Additionally, in La Gomera, Tenerife, and Gran Canaria, males presented a marked increase in LEH prevalence between the ages of 4–5 and 5–6 years, whereas this trend was less evident among females. This observation aligns with the “female buffering hypothesis” or “male vulnerability hypothesis” (Stinson 1985). This hypothesis suggests that males, owing to their more rapid and extended growth periods, greater energy allocation to the brain and lean muscle development, and relatively lower levels of adipose tissue than females do, are more susceptible to environmental or nutritional stress during early life (Bogin 1999; Eveleth and Tanner 1991). Consequently, this could result in a marked increase in the number of LEHs in males across almost all indigenous populations.

However, interpretations of sex‐based differences in LEH frequencies must be interpreted also consider the osteological paradox (Wood et al. 1992): LEH can only be observed in individuals who survived the stress episode long enough for the defect to form and for the tooth to be recovered archeologically. Therefore, it is possible that females exposed to similar or even greater levels did not survive and thus are underrepresented in the adult sample (King et al. 2005). While this survival bias remains a valid concern in paleopathological studies, in the present case, it is likely to have had a limited effect. Chi‐square analyses did not reveal significant sex differences in the demographic composition of the studied sample, suggesting that male and female individuals were equally represented in the age groups analyzed. Therefore, the hypothesis that higher female mortality led to lower observed LEH prevalence in females appears unlikely.

An alternative explanation is that females may have received preferential care during early life, which could have buffered them against the physiological stressors that lead to LEH formation. Although direct archeological evidence for such practices in childhood is lacking, some indirect support comes from ethnohistorical sources referenced by Santana et al. (2019). In their analysis of a pregnant woman's burial in Gran Canaria, the authors suggest enhanced care for women of reproductive age, based on the funerary context and early colonial accounts describing special attention to pregnant women in indigenous Canarian society. While these practices refer to adults rather than children, they raise the possibility that cultural norms may have included gendered forms of care that could have extended to female children. Nonetheless, given the lack of sex‐based differences in mortality in our sample and the absence of systematic archeological evidence for differential treatment during childhood, this explanation remains speculative. Biological sex differences in developmental stress susceptibility, therefore, continue to offer the most parsimonious explanation for the observed LEH patterns.

Furthermore, and interestingly, the sex differences in developmental stress due to a possible “male vulnerability” were not observed in La Palma, likely because of its subsistence strategy. This finding underscores the importance of considering the high energy requirements during the first 6 years of life. In the first 5 years, these requirements are largely met by individuals' fat stores (Kuzawa 1998); from the age of 5 years, these fat reserves reach their lowest point (Leonard et al. 2007), increasing the susceptibility of children to variations in food intake. A diet predominantly based on marine, livestock, and wild plant sources, such as that in La Palma, potentially provides children with better energy reserves (more fat stores). This could be associated with a reduced impact of malnutrition, particularly affecting males. Therefore, the dietary patterns in La Palma might offer a protective effect against the development of LEH, especially in male children, because better nutritional status is facilitated by these dietary sources.

In addition to the type of food that children consume, other factors may play a role in why male children from La Palma do not exhibit greater vulnerability than females do. One key aspect is the stage of dental development in children. At this age, children possess only their deciduous teeth and have not yet developed an adult chewing cycle, which limits their ability to consume foods in the same manner as adults do (Bogin 1999; Scott and Halcrow 2017). Foods are often made child friendly by boiling, steaming, toasting, or converting them into semisolid forms, such as through premastication or mashing (Sellen and Smay 2001). Zooarchaeological data from La Palma indicate a culinary preference for boiling meat to create soups, which contrasts with roasting at the other islands (Pais 1996). Notably, the European ethnohistorical textual records indicate that the predominant components of the indigenous diet in La Palma consisted of meat or milk soups supplemented with fern roots and/or *amagante* seeds (a plant endemic to the region and akin to rockrose), as well as caprine and pig meat: “The food they used instead of bread were fern roots and amagante grain […] This grain was harvested at the right time, dried, ground in hand mills, and then stored to be eaten with meat broth or milk, and they also sustained themselves with sheep and goat meat, which they called teguevite, and with pork, which they called atiuavina” (Abreu Galindo 1977, 195).

This cooking method helps children consume nutrient‐rich parts of meat, such as bone marrow and adipose tissue, which are essential for growth and development (Kuzawa 1998). Cooking techniques such as steaming, toasting, or roasting might lead to fat loss, whereas boiling results in the retention of these vital nutrients (Carmody and Wrangham 2009). Hence, La Palma's cooking methods, especially boiling meat to preserve fat, likely offered essential growth nutrients, influencing the LEH prevalence patterns in males during development. Notably, dental macrowear analyses and ethnohistorical textual records indicate the consumption of fern roots in La Palma as a famine food (Morquecho Izquier et al. 2025). Fern roots likely provide energy and other nutrients during periods of food scarcity. Additional insights might be obtained from alternative data sets in the future, including dental microwear analysis of deciduous teeth or changes in mandibular shape and cross‐sectional properties (García‐González et al. 2019; von Cramon‐Taubadel 2011).

Further evidence from Gran Canaria supports the role of diet: both males and females presented an increase in LEH prevalence starting at age 4, which is likely linked to the lower fat content of agricultural diets than of diets richer in animal and marine resources. Notably, incremental dentine isotopic analysis in Gran Canaria's population indicated that the weaning process occurred between the ages of 2 and 4 years (Sánchez‐Cañadillas et al. 2023). Fat plays a critical role in early childhood in terms of supporting energy metabolism and immune function, and its scarcity could have amplified nutritional stress, resulting in increased LEH prevalence in both sexes.

Taken together, our findings align more closely with the plasticity/constraint hypothesis, as LEH prevalence reflects how populations adapt their growth and survival strategies to environmental and nutritional constraints. Although some elements of the data could support the Predictive Hypothesis, for example, individuals surviving stress events exhibit resilience. Overall patterns suggest that plasticity in response to environmental and subsistence constraints played a more significant role in shaping LEH prevalence across the Canary Islands.

We are aware that there are some methodological limitations in our study, many of which are inherent to studies of archeological populations. The first of these limitations is related to the nature of the sample itself, with an important bias in the representation of each tooth. This limitation may restrict the interpretations that can be made regarding comparisons among different indigenous populations. Second, the use of a macroscopic method may influence our results, as features interpreted as evidence of growth disruption microscopically are not always consistently identified with the naked eye. This highlights the level of consistency in the number and timing of defects detected through microscopic vs. macroscopic approaches (Hassett 2014). Nonetheless, this limitation would affect all populations equally, ensuring comparability. The primary factor that could impact the results is dental wear; however, as we have demonstrated, differences in dental wear appear to have minimal influence on the comparisons between populations. Despite these limitations, this study provides valuable insight precisely because it is one of the few to assess LEH prevalence in molars, offering a much‐needed point of comparison for future research in which posterior teeth are more frequently preserved than anterior ones. As such, it highlights the importance of adapting analytical expectations to the anatomical and taphonomic limitations of archeological samples.

## 5 Conclusions

The detailed analysis of LEH incidence, its age at appearance, and its relationship with age at death reveals significant differences among indigenous populations that are linked to subsistence strategies. These differences align well with the plasticity/constraint hypothesis. Gran Canaria and La Palma serve as key examples. In Gran Canaria, reliance on agriculture increased exposure to stress, but adaptations such as food storage and irrigation helped buffer its effects. Nevertheless, the inherent constraints of the agricultural system led to recurring episodes of stress. In contrast, in La Palma, a subsistence strategy focused on wild plants, marine, and animal resources, along with nutrient‐preserving cooking methods, demonstrates a plastic adaptive response to mitigate stress. These practices notably reduced the impact of stress events, particularly among males.

## Author Contributions

**Aarón Morquecho Izquier:** conceptualization (supporting), data curation (lead), formal analysis (equal), investigation (equal), methodology (equal), visualization (equal), writing – original draft (equal), writing – review and editing (equal). **Rebeca García‐González:** conceptualization (lead), formal analysis (equal), investigation (equal), methodology (equal), visualization (equal), writing – original draft (equal), writing – review and editing (equal). **Jonathan Santana:** conceptualization (supporting), funding acquisition (lead), methodology (equal), project administration (lead), visualization (equal), writing – original draft (equal), writing – review and editing (equal).

## Ethics Statement

This study received approval from the Cultural Heritage Office of the Government of the Canary Islands, the authority responsible for overseeing the heritage of the indigenous communities of the archipelago. Additionally, we obtained the necessary permission from the different island archeological museums. The analysis of human remains housed at the Musée de l'Homme was conducted under the auspices of this institution's governing commission.

## Supporting information


**Data S1:** Supporting Information.

## Data Availability

The data that support the findings of this study are available in Supporting Information S1.
