# Supplementary material for: Stress, Life History, and Linear Enamel Hypoplasia: Insights From the Indigenous Populations of the Canary Islands
Source: Am J Biol Anthropol. 2025 Aug 29;188(1):e70116. doi: 10.1002/ajpa.70116 (PMC12395211; doi:10.1002/ajpa.70116)
Supplement: Supplementary file 1 — Data S1: Supporting Information. [file AJPA-188-e70116-s001.docx]

**Table S1. Dental macrowear severity in first and second molars among Indigenous population**

| **Population** | **Light dental macrowear** | | **Medium dental macrowear** | | **Heavy dental macrowear** | | |
| --- | --- | --- | --- | --- | --- | --- | --- |
|  | **First molar** | **Second molar** | **First molar** | **Second molar** | **First molar** | **Second molar** |  |
| **El Hierro** | 2.5% (4/160) | 7.5% (13/172) | 12.5% (20/160) | 41.8% (72/172) | 85% (136/160) | 50.5% (87/172) |  |
| **La Palma** | 6.0% (2/33) | 23.5% (8/34) | 24.2% (8/33) | 26.5% (9/34) | 69.7% (23/33) | 50.0% (17/34) |  |
| **La Gomera** | 2.9% (3/104) | 12.8% (15/117) | 39.4% (41/104) | 63.2% (74/117) | 57.7% (60/104) | 23.9% (28/117) |  |
| **Tenerife** | 12.8% (6/47) | 25.0% (13/52) | 19.1% (9/47) | 46.1% (25/52) | 68.1% (32/47) | 28.8% (15/52) |  |
| **Gran Canaria** | 9.7% (26/269) | 32.8% (79/241) | 33.4% (90/269) | 48.1% (116/241) | 19.7% (53/269) | 19.1% (46/241) |  |
| **Fuerteventura** | 8.3% (5/60) | 28.8% (15/52) | 73.3% (44/60) | 65.4% (34/52) | 18.3% (11/60) | 5.7% (3/52) |  |

**Table S2. Results from statistical comparisons between Indigenous population in light dental macrowear category for first and second molars.**

|  | **El Hierro** | **La Palma** | **La Gomera** | **Tenerife** | **Gran Canaria** | **Fuerteventura** |
| --- | --- | --- | --- | --- | --- | --- |
| **El Hierro** |  | 0.40*, p=0.27 | 0.87*, p=1.00 | **0.17*, p=0.01** | **7.75, p=0.00** | 0.28, p=0.11 |
| **La Palma** | **0.26*, p=0.01** |  | 2.15*, p=0.59 | 0.44*, p=0.45 | 0.60, p=0.75 | 0.71*, p=1.00 |
| **La Gomera** | 2.20, p=0.13 | 2.33, p=0.12 |  | **0.20*, p=0.02** | **4.80, p=0.02** | 0.32*, p=0.14 |
| **Tenerife** | **11.83, p=0.00** | 0.02 p=0.87 | **3.86, p=0.04** |  | 1.36*, p=0.59 | 1.60*, p=0.52 |
| **Gran Canaria** | **36.87, p=0.00** | 1.17, p=0.27 | **16.20, p=0.00** | **3.86, p=0.04** |  | 0.10, p=0.74 |
| **Fuerteventura** | **16.54, p=0.00** | 0.29 p=0.58 | **6.33, p=0.01** | **16.20, p=0.00** | 0.30, p=0.58 |  |

*Above the diagonal differences between populations in light dental macrowear for first molar. Below the diagonal differences between populations in light dental macrowear for second molars. Chi-square or odds-ratio (*) values and their probabilities associated.*

**Table S3. Results from statistical comparisons between Indigenous population in medium dental macrowear category for first and second molars.**

|  | **El Hierro** | **La Palma** | **La Gomera** | **Tenerife** | **Gran Canaria** | **Fuerteventura** |
| --- | --- | --- | --- | --- | --- | --- |
| **El Hierro** |  | 0.44*, p=0.10 | **25.71, p=0.00** | 1.33, p=0.24 | **23.11, p=0.00** | **78.28, p=0.00** |
| **La Palma** | 2.81, p=0.09 |  | 2.51, p=0.11 | 0.30, p=0.58 | 1.13, p=0.28 | **20.81, p=0.00** |
| **La Gomera** | **12.74, p=0.00** | **14.39, p=0.00** |  | **6.00, p=0.01** | 1.17, p=0.27 | **17.52, p=0.00** |
| **Tenerife** | 0.30 p=0.58 | 3.36, p=0.06 | **4.31, p=0.03** |  | **3.80, p=0.05** | **30.95, p=0.00** |
| **Gran Canaria** | 1.59, p=0.20 | **5.63, p=0.01** | **7.22, p=0.00** | 0.06, p=0.79 |  | **32.32, p=0.00** |
| **Fuerteventura** | **8.86, p=0.00** | **12.45 p=0.00** | 0.07, p=0.78 | **3.89, p=0.04** | **5.09, p=0.02** |  |

*Above the diagonal differences between populations in medium dental macrowear for first molar. Below the diagonal differences between populations in medium dental macrowear for second molars. Chi-square or odds-ratio (*) values and their probabilities associated.*

**Table S4. Results from statistical comparisons between Indigenous population in heavy dental macrowear category for first and second molars.**

|  | **El Hierro** | **La Palma** | **La Gomera** | **Tenerife** | **Gran Canaria** | **Fuerteventura** |
| --- | --- | --- | --- | --- | --- | --- |
| **El Hierro** |  | **4.41, p=0.03** | **6.79, p=0.00** | **36.09, p=0.00** | **21.05, p=0.00** | **87.47, p=0.00** |
| **La Palma** | 0.00, p=0.95 |  | 1.51, p=0.21 | 0.02, p=0.87 | 1.98, p=0.15 | **24.21, p=0.00** |
| **La Gomera** | **20.64, p=0.00** | **8.55, p=0.00** |  | 1.46, p=0.22 | 0.02, p=0.88 | **24.00, p=0.00** |
| **Tenerife** | **7.60, p=0.00** | **3.93, p=0.04** | 0.45, p=0.49 |  | 2.07, p=0.15 | **27.13, p=0.00** |
| **Gran Canaria** | **45.59, p=0.00** | **16.12, p=0.00** | 1.12, p=0.28 | 2.47, p=0.11 |  | **29.15, p=0.00** |
| **Fuerteventura** | **33.36, p=0.00** | **22.53, p=0.00** | **7.92, p=0.00** | **9.67, p=0.00** | **5.44, p=0.01** |  |

*Above the diagonal differences between populations in heavy dental macrowear for first molar. Below the diagonal differences between populations in heavy dental macrowear for second molars. Chi-square or odds-ratio (*) values and their probabilities associated.*

**Table S5. Summary statistics of crown heights of first molars of each Indigenous population.**

| **Population** | **Upper first molar** | | | **Lower first molar** | | |
| --- | --- | --- | --- | --- | --- | --- |
|  | **Females** | **Males** | **Total** | **Females** | **Males** | **Total** |
| **El Hierro** | 5.07 + 0.77 (6) | 5.48 + 0.64 (15) | 5.32 + 0.70 (22) | 4.88 + 1.40 (3) | 4.49 +0.82 (3) | 4.69 + 1.04 (6) |
| **La Palma** | 6.18 + 0.48 (2) | 4.92 + 1.10 (2) | 5.38 + 0.78 (7) | 4.59 + 1.28 (2) | 5.24 + 0.75 (6) | 4.01 + 0.83 (12) |
| **La Gomera** | 5.88 + 0.28 (4) | 5.28 + 0.69 (7) | 5.36 + 0.79 (18) | 4.00 + 0.66 (6) | 4.89 + 0.74 (5) | 4.40 + 0.86(14) |
| **Tenerife** | 5.68 + 0.56 (4) | 4.87 +1.38 (6) | 5.25 + 1.05 (12) | --- | 5.03 (1) | 5.03 (1) |
| **Gran Canaria** | 6.04 + 0.51 (18) | 6.57 + 0.55 (14) | 6.29 + 0.56 (39) | 6.01 + 0.22 (4) | 5.66 + 0.56 (2) | 5.92 + 0.41 (11) |
| **Fuerteventura** | 6.12 + 0.42 (2) | 5.39 + 0.50 (8) | 5.49 + 0.55 (11) | 5.47 + 1.03 (4) | 5.12 + 1.03 (7) | 5.14 + 0.98 (12) |
| **Total** | 5.83 + 0.63 (36) | 5.64 + 0.92 (52) | 5.69 + 0.83 (109) | 4.93 + 1.10(19) | 5.06 + 0.81 (24) | 5.03 + 0.95 (56) |

*Mean + standard deviation (N). The total N is calculated considering individuals sexed as males, females and those undetermined.*

**Table S6. Summary statistics of crown heights of second molars of each Indigenous population.**

| **Population** | **Upper second molar** | | | **Lower second molar** | | |
| --- | --- | --- | --- | --- | --- | --- |
|  | **Females** | **Males** | **Total** | **Females** | **Males** | **Total** |
| **El Hierro** | 5.37 + 0.83 (7) | 5.72 + 0.69 (18) | 5.57 + 0.78 (27) | 5.43 + 0.74 (5) | 5.30 + 0.51 (9) | 5.43 + 0.27 (17) |
| **La Palma** | 4.76 + 1.22 (2) | 5.19 + 0.87 (8) | 5.09 + 0.78 (13) | 5.48 (1) | 5.18 + 1.27 (8) | 5.34 + 1.10 (11) |
| **La Gomera** | 5.41 + 0.57 (10) | 5.52 + 0.48 (12) | 5.51 +0.52 (30) | 5.13 + 0.75 (4) | 5.40 + 0.59 (8) | 5.39 + 0.78 (17) |
| **Tenerife** | 5.67 + 0.36 (7) | 5.13 + 0.37 (9) | 5.40 + 0.53 (19) | 5.31 (1) | 5.21 (1) | 5.26 + 0.07 (2) |
| **Gran Canaria** | 5.99 + 0.53 (30) | 6.24 + 0.69 (26) | 6.13 + 0.60 (73) | 5.05 + 0.64 (17) | 5.49 + 0.77 (17) | 5.34 + 0.76 (43) |
| **Fuerteventura** | 5.97 + 0.20 (3) | 5.01 + 0.43 (8) | 5.21 + 0.60 (12) | 5.40 + 0.88 (6) | 5.69 + 0.53 (6) | 5.54 + 0.71 (12) |
| **Total** | 5.74 + 0.64 (59) | 5.67 + 0.77 (81) | 5.71 + 0.72 (174) | 5.19 + 0.69 (34) | 5.41 + 0.77 (49) | 5.37 + 0.77 (102) |

*Mean + standard deviation (N). The total N is calculated considering individuals sexed as males, females and those undetermined.*

**Table S7. Results from statistical analysis among population.**

|  | **El Hierro** | **La Palma** | **La Gomera** | **Tenerife** | **Gran Canaria** | **Fuerteventura** |
| --- | --- | --- | --- | --- | --- | --- |
| **El Hierro** |  | 2.57*, p=0.10 | 0.20, p=0.65 | 2.73, p=0.10 | 0.65, p=0.42 | 0.50, P=0.48 |
| **La Palma** |  |  | 4.00*, p=0.08 | **0.19*, p=0.01** | **0.30*, p=0.03** | 0.,27*, p=0.08 |
| **La Gomera** |  |  |  | 1.44, p=0.23 | 0.04, p=.0.83 | 0.14, p=0.71 |
| **Tenerife** |  |  |  |  | 1.40, p=0.24 | 0.36, p=0.54 |
| **Gran Canaria** |  |  |  |  |  | 0.07, p=0.79 |
| **Fuerteventura** |  |  |  |  |  |  |

** Denotes odds-ratio values*

**Table S8. Results from statistical comparisons between sexes of the same population**

| **Population** | **Chi-square/Odds-ratio** | **Probability** |
| --- | --- | --- |
| El Hierro | 0.79 | 0.37 |
| La Palma | 0.51* | 1.00 |
| La Gomera | 1.00* | 1.00 |
| Tenerife | 1.19* | 1.00 |
| Gran Canaria | 4.64 | ***0.04*** |
| Fuerteventura | 2.15 | 0.62 |

** Denotes odds-ratio values*

**Table S9. Results from statistical comparisons between age groups from 17-25 years and from 26-35 years by Indigenous population.**

| **Population** | **Chi-square/Odds-ratio** | **Probability** |
| --- | --- | --- |
| El Hierro |  |  |
| La Palma |  |  |
| La Gomera |  |  |
| Tenerife |  |  |
| Gran Canaria |  |  |
| Fuerteventura |  |  |

**Table S10. Results from statistical analysis between populations in the number of LEH formed during the two first age-categories established in this study.**

|  | **El Hierro** | **La Palma** | **La Gomera** | **Tenerife** | **Gran Canaria** | **Fuerteventura** |
| --- | --- | --- | --- | --- | --- | --- |
| **El Hierro** |  | 1.16*, p=1 | 0.16, p=0.69 | 0.91*, p=1 | 0.56, p=0.45 | 0.67*, p=0.50 |
| **La Palma** | 0.66*, p=0.72 |  | 0.70*, p=0.75 | 0.78*, p=1 | 0.61*, p=0.57 | 0.58*, p=0.69 |
| **La Gomera** | 1.09*, p=1 | 1.65*, p=0.48 |  | 1.12*, p=1 | 0.08, p=0.88 | 0.82*, p=0.74 |
| **Tenerife** | 0.68*, p=0.72 | 1.04*, p=1 | 0.62*, p=0.49 |  | 0.78*, p=0.79 | 0.73*, p=0.71 |
| **Gran Canaria** | 3.18, p=0.07 | 0.65*, p=0.60 | 0.39*, p=0.08 | 0.63*, p=0.60 |  | 0.93*, p=1 |
| **Fuerteventura** | . Inf, p=0.33 | .Inf, p=0.14 | .Inf, p=0.32 | .Inf, p=0.15 | .Inf, p=0.04 |  |

*Above the diagonal, the Chi-square or odds-ratio (*) values and their associated probabilities result from comparisons between populations for the number of LEH formed between 1 and 2 years. Below the diagonal, the Chi-square or odds-ratio () values and their associated probabilities result from comparisons between populations for the number of LEH formed between 2 and 3 years.*

**Table S11. Results from statistical analysis between populations in the number of LEH formed during the two older age-categories established in this study.**

|  | **El Hierro** | **La Palma** | **La Gomera** | **Tenerife** | **Gran Canaria** | **Fuerteventura** |
| --- | --- | --- | --- | --- | --- | --- |
| **El Hierro** |  | 1.16*, p=1 | 0.16, p=0.69 | 0.91*, p=1 | 0.56, p=0.45 | 0.67*, p=0.50 |
| **La Palma** | 0.66*, p=0.72 |  | 0.70*, p=0.75 | 0.78*, p=1 | 0.61*, p=0.57 | 0.58*, p=0.69 |
| **La Gomera** | 1.09*, p=1 | 1.65*, p=0.48 |  | 1.12*, p=1 | 0.08, p=0.88 | 0.82*, p=0.74 |
| **Tenerife** | 0.68*, p=0.72 | 1.04*, p=1 | 0.62*, p=0.49 |  | 0.78*, p=0.79 | 0.73*, p=0.71 |
| **Gran Canaria** | 3.18, p=0.07 | 0.65*, p=0.60 | 0.39*, p=0.08 | 0.63*, p=0.60 |  | 0.93*, p=1 |
| **Fuerteventura** | . Inf, p=0.33 | .Inf, p=0.14 | .Inf, p=0.32 | .Inf, p=0.15 | .Inf, p=0.04 |  |

*Above the diagonal, the Chi-square or odds-ratio (*) values and their associated probabilities result from comparisons between populations for the number of LEH formed between 4 and 5 years. Below the diagonal, the Chi-square or odds-ratio () values and their associated probabilities result from comparisons between populations for the number of LEH formed between 5 and 6 years.*

**Table S12. Sexual differences by age group and population.**

| **Population** | **Age groups** | | | |
| --- | --- | --- | --- | --- |
|  | **1-2** | **2-3.5** | **4-5** | **5-6** |
| **El Hierro** | 1.33*, p=1 | 2.97*, p=1 | 1*, P=1 | 1.16*, p=1 |
| **La Palma** | 1.97*, p=1 | 0*, p=1 | 0*, p=1 | .Inf*, p=0.49 |
| **La Gomera** | 2.97*, p=0.62 | 3.95*, p=0.36 | 0.25*, p=0.36 | **.Inf*, p=0.01** |
| **Tenerife** | 0.33*, p=0.61 | 1*, p=1 | 0*, p=0.23 | **.Inf*, p=0.01** |
| **Gran Canaria** | 0.55, p=1.50 | 0.23, p=0.63 | **6.94*, p=0.05** | 2.84, p=0.09 |
| **Fuerteventura** | 0.34*, p=0.60 | 0*, p=1 | 0*, p=1 | 0*, p=1 |

*Chi-square or odds-ratio (*) values and their probabilities associated.*

**Table S13. Differences in the number of LEH formed during each of age categories established in this study by population and sex.**

| **Population** | **Sex** | **AG 1/ AG2** | **AG 1/ AG3** | **AG 1/ AG4** | **AG 2/ AG3** | **AG 2/ AG4** | **AG 3/ AG4** |
| --- | --- | --- | --- | --- | --- | --- | --- |
| **El Hierro** | **Female** | 2.97*, p=0.62 | 1.93*, p=1 | 0.33*, p=0.16 | 0.65*, p=1 | **0.11*, p=0.04** | 0.17*, p=0.11 |
|  | **Male** | 1.33*, p=1 | 2.57*, p=0.65 | 0.37*, p=0.19 | 1.93*, p=1 | 0.28*, p=0.09 | **0.14*, p=0.05** |
| **La Palma** | **Female** | .Inf*, p=1 | 1.12, p=1 | .Inf*, p=0.48 | 0*, p=1 | 0*, p=1 | .Inf*, p=1 |
|  | **Male** | .Inf*, p=0.49 | .Inf*, p=0.24 | 1.12*, p=1 | 0*, p=1 | 0*, p=0.49 | 0*, p=0.49 |
| **La Gomera** | **Female** | 0*, p=1 | 0.27*, p=0.37 | .Inf*, p=0.49 | 0.27*, p=0.37 | .Inf*, p=0.49 | .Inf*, p=0.12 |
|  | **Male** | 0.75*, p=1 | 3.20*, p=0.36 | 0.46*, p=0.33 | 4.26*, p=0.21 | 0.62*, p=0.54 | **0.15*, p=0.05** |
| **Tenerife** | **Female** | 0.50*, p=1 | 0.39*, p=0.62 | .Inf*, p=0.47 | 0.77*, p=1 | .Inf*, p=0.23 | .Inf*, p=0.11 |
|  | **Male** | 1.49*, p=1 | .Inf*, p=0.11 | 0.50*, p=0.49 | .Inf*, p=0.23 | 0.33*, p=0.28 | **0*, p=0.01** |
| **Gran Canaria** | **Female** | 0.07, p=0.79 | 5.14*, p=0.13 | 0.61, p=0.43 | 5.99*, p=0.07 | 0.75, p=0.60 | **0.13*, p=0.03** |
|  | **Male** | 0, p=1 | 0.04, p=0.84 | 3.32, p=0.07 | 0.04, p=0.84 | 3.32, p=0.07 | **3.67, p=0.05** |
| **Fuerteventura** | **Female** | .Inf*, p=0.23 | 3.08*, p=0.61 | 3.08*, p=0.61 | 0*, p=1 | 0*, p=1 | 1*, p=1 |
|  | **Male** | .Inf*, p=1 | 1.05*, p=1 | .Inf*, p=1 | 0*, p=1 | 0*, p=1 | .Inf*, p=1 |

*AG1: age group 1(1-2 years), AG1: age group 2 (2-3.5 years), AG3: age group 3 (4-5 years), AG4: age group 4 (5-6 years). Chi-square or odds-ratio (*) values and their probabilities associated.*

**Table S14. Results derived from statistical comparisons among populations with a single stress episode.**

|  | **El Hierro** | **La Palma** | **La Gomera** | **Tenerife** | **Gran Canaria** | **Fuerteventura** |
| --- | --- | --- | --- | --- | --- | --- |
| **El Hierro** |  | 2.93*, p=0.63 | 1.48, p=0.22 | 0.35, p=0.55 | **3.81, p=0.05** | 1.30*, p=0.74 |
| **La Palma** |  |  | 0.65*, p=1 | 0.48*, p=1 | 0.77*, p=1 | 0.46*, p=1 |
| **La Gomera** |  |  |  | 0.25, p=0.61 | 0.10, p=0.74 | 0.69*, p=0.70 |
| **Tenerife** |  |  |  |  | 0.82, p=0.39 | 0.94*, p=1 |
| **Gran Canaria** |  |  |  |  |  | 0.57*, p=0.46 |
| **Fuerteventura** |  |  |  |  |  |  |

*Chi-square or odds-ratio (*) values and their probabilities associated.*

**Table S15. Results derived from statistical comparisons among populations with two stress episodes.**

|  | **El Hierro** | **La Palma** | **La Gomera** | **Tenerife** | **Gran Canaria** | **Fuerteventura** |
| --- | --- | --- | --- | --- | --- | --- |
| **El Hierro** |  | 0.43*, p=0.58 | 1.39, p=0.24 | 0.68*, p=0.71 | 2.31, p=0.13 | 0.50*, p=0.40 |
| **La Palma** |  |  | 1.12*, p=1 | 1.59*, p=0.63 | 1.04*, p=1 | 1.15*, p=1 |
| **La Gomera** |  |  |  | 1.43*, p=0.73 | 0.02, p=0.88 | 1.03*, p=1 |
| **Tenerife** |  |  |  |  | 0.49, p=0.48 | 0.73*, p=1 |
| **Gran Canaria** |  |  |  |  |  | 1.11*, p=1 |
| **Fuerteventura** |  |  |  |  |  |  |

*Chi-square or odds-ratio (*) values and their probabilities associated.*

**Table S16. Results derived from statistical comparisons among populations with three stress episodes.**

|  | **El Hierro** | **La Palma** | **La Gomera** | **Tenerife** | **Gran Canaria** | **Fuerteventura** |
| --- | --- | --- | --- | --- | --- | --- |
| **El Hierro** |  | 0.18*, p=0.31 | 0.38*, p=0.58 | 0.55*, p=1 | 0.21*, p=0.16 | .Inf*, p=1 |
| **La Palma** |  |  | 2.16*, p=0.50 | 3.01*, p=0.47 | 1.27*, p=1 | .Inf*, p=0.42 |
| **La Gomera** |  |  |  | 1.43*, p=1 | 0.57*, p=0.72 | .Inf*, p=1 |
| **Tenerife** |  |  |  |  | 0.39*, p=0.67 | .Inf*, p=1 |
| **Gran Canaria** |  |  |  |  |  | .Inf*, p=0.58 |
| **Fuerteventura** |  |  |  |  |  |  |

*Chi-square or odds-ratio (*) values and their probabilities associated.*
